# Comparability of automated drusen volume measurements in age-related macular degeneration: a MACUSTAR study report

**DOI:** 10.1038/s41598-022-26223-w

**Published:** 2022-12-19

**Authors:** Davide Garzone, Jan Henrik Terheyden, Olivier Morelle, Maximilian W. M. Wintergerst, Marlene Saßmannshausen, Steffen Schmitz-Valckenberg, Maximilian Pfau, Sarah Thiele, Stephen Poor, Sergio Leal, Frank G. Holz, Robert P. Finger, MACUSTAR Consortium, H. Agostini, L. Altay, R. Atia, F. Bandello, P. G. Basile, C. Behning, M. Belmouhand, M. Berger, A. Binns, C. J. F. Boon, M. Böttger, C. Bouchet, J. E. Brazier, T. Butt, C. Carapezzi, J. Carlton, A. Carneiro, A. Charil, R. Coimbra, M. Cozzi, D. P. Crabb, J. Cunha-Vaz, C. Dahlke, L. de Sisternes, H. Dunbar, E. Fletcher, C. Francisco, M. Gutfleisch, R. Hogg, C. B. Hoyng, A. Kilani, J. Krätzschmar, L. Kühlewein, M. Larsen, Y. T. E. Lechanteur, U. F. O. Luhmann, A. Lüning, I. Marques, C. Martinho, G. Montesano, Z. Mulyukov, M. Paques, B. Parodi, M. Parravano, S. Penas, T. Peters, T. Peto, S. Priglinger, D. Rowen, G. S. Rubin, J. Sahel, C. Sánchez, O. Sander, M. Schmid, H. Schrinner-Fenske, J. Siedlecki, R. Silva, A. Skelly, E. Souied, G. Staurenghi, L. Stöhr, D. J. Taylor, A. Tufail, M. Varano, L. Vieweg, L. Wintergerst, A. Wolf, N. Zakaria

**Affiliations:** 1 Department of Ophthalmology, University Hospital Bonn, Ernst-Abbe Str. 2, 53127 Bonn, Germany; 2 Population Health Sciences, German Center for Neurodegenerative Diseases (DZNE), Bonn, Germany; 3 B-IT and Institute of Informatics, University of Bonn, Bonn, Germany; 4 John A. Moran Eye Center, University of Utah, Salt Lake City, USA; 5 Ophthalmology, Novartis Institutes for Biomedical Research, Cambridge, MA USA; 6 Bayer Pharmaceuticals, Berlin, Germany; 7 Department of Ophthalmology, Universitaetsklinikum Freiburg (UKLFR), University of Freiburg, Freiburg, Germany; 8 Department of Ophthalmology, University Hospital of Cologne, Cologne, Germany; 9 Quinze-Vingts National Ophthalmology Hospital, UPMC-Sorbonne Université, Paris, France; 10 Department of Ophthalmology, University Vita Salute-Scientific Institute of San Raffaele, Milan, Italy; 11 AIBILI Association for Innovation and Biomedical Research on Light and Image (AIBILI), Coimbra, Portugal; 12 Institute for Medical Biometry, Informatics and Epidemiology, University of Bonn, Bonn, Germany; 13 Department of Ophthalmology, Rigshospitalet-Glostrup, Copenhagen University, Glostrup, Denmark; 14 City University London, London, UK; 15 Department of Ophthalmology, Leiden University Medical Center, Leiden, The Netherlands; 16 BAYER AG, Leverkusen, Germany; 17 Novartis Pharma AG, Basel, Switzerland; 18 University of Sheffield, Sheffield, UK; 19 University College London (UCL), London, UK; 20 Fondation Voir et Etendre, Paris, France; 21 Department of Ophthalmology, Porto Medical School, Centro Hospitalar de Sao Joao EPE (Hospital Sao Joao), Porto, Portugal; 22 Department of Ophthalmology Luigi Sacco Hospital, University of Milan, Milan, Italy; 23 Carl Zeiss Meditec, AG, Jena, Germany; 24 Clinical Trial Unit, Department of Ophthalmology, Gloucestershire Hospitals NHS Foundation Trust, Cheltenham, UK; 25 Department of Ophthalmology, St. Franziskus Hospital, Münster, Germany; 26 Ophthalmology and Vision Sciences, The Queen’s University and Royal Group of Hospitals Trust, Belfast, Northern Ireland UK; 27 Stichting Katholieke Universiteit/Radboud University Medical Center (SRUMC), Nijmegen Medical Center, Radbound University, Nijmegen, The Netherlands; 28 Department of Ophthalmology, University of Ulm, Ulm, Germany; 29 STZ Biomed and STZ Eyetrial at the Center of Ophthalmology, University Hospital Tuebingen, Tübingen, Germany; 30 F. Hoffmann-La Roche Ltd, Basel, Switzerland; 31 G. B. Bietti Eye Foundation-IRCCS, Rome, Italy; 32 Ludwig-Maximilians-Universitaet Muenchen (LMU), University Eye Hospital, Munich, Germany; 33 European Clinical Research Infrastructure Network (ECRIN), Paris, France; 34 Centre Hospitalier Intercommunal de Creteil (HIC), Centre Hospitalier Creteil, University Eye Clinic, Paris, France; 35 Moorfields Eye Hospital NHS Foundation Trust (MBRC), London, UK

**Keywords:** Biomarkers, Medical research, Risk factors

## Abstract

Drusen are hallmarks of early and intermediate age-related macular degeneration (AMD) but their quantification remains a challenge. We compared automated drusen volume measurements between different OCT devices. We included 380 eyes from 200 individuals with bilateral intermediate (iAMD, n = 126), early (eAMD, n = 25) or no AMD (n = 49) from the MACUSTAR study. We assessed OCT scans from Cirrus (200 × 200 macular cube, 6 × 6 mm; Zeiss Meditec, CA) and Spectralis (20° × 20°, 25 B-scans; 30° × 25°, 241 B-scans; Heidelberg Engineering, Germany) devices. Sensitivity and specificity for drusen detection and differences between modalities were assessed with intra-class correlation coefficients (ICCs) and mean difference in a 5 mm diameter fovea-centered circle. Specificity was > 90% in the three modalities. In eAMD, we observed highest sensitivity in the denser Spectralis scan (68.1). The two different Spectralis modalities showed a significantly higher agreement in quantifying drusen volume in iAMD (ICC 0.993 [0.991–0.994]) than the dense Spectralis with Cirrus scan (ICC 0.807 [0.757–0.847]). Formulae for drusen volume conversion in iAMD between the two devices are provided. Automated drusen volume measures are not interchangeable between devices and softwares and need to be interpreted with the used imaging devices and software in mind. Accounting for systematic difference between methods increases comparability and conversion formulae are provided. Less dense scans did not affect drusen volume measurements in iAMD but decreased sensitivity for medium drusen in eAMD.

**Trial registration:** ClinicalTrials.gov NCT03349801. Registered on 22 November 2017.

## Introduction

Age-related macular degeneration (AMD) continues to be a major cause of visual impairment and in order to better predict the risk of disease progression and outcomes, better phenotyping and thus AMD staging is needed^1,2^. The Beckmann classification is widely adopted for AMD staging; it is based on maximal drusen diameter cut-offs measured on color fundus photography (CFP), with measurements between 63 and 125 µm defining early AMD (eAMD) and larger than 125 µm defining intermediate AMD (iAMD)^3^. Using solely two-dimensional CFP for AMD disease staging is somewhat outdated; semi-quantitative assessment of multi-modal imaging has become a more common approach for retinal experts in day to day clinical routine. In particular, optical coherence tomography (OCT) lends itself to biomarker quantification, i.e. the calculation of drusen load using three-dimensional information for the quantification of drusen volume^4^. Previous studies observed an association between larger drusen volumes and an increased risk of AMD progression^5,6^, while drusen volume regression can precede conversion to late AMD lesions^7^. Drusen volume might be also more precisely measurable and repeatable than drusen area^5,8^, thus making it a promising biomarker and structural endpoint in AMD. OCT also allows for accurate assessment of reticular pseudodrusen (RPD), which are not included in the Beckmann classification but have emerged as an important biomarker of AMD severity and progression risk^9^.

Automated algorithms for drusen volume quantification are available including a software for the high definition-OCT Cirrus (Carl Zeiss Meditec, Dublin, CA), achieving approval by the Food and Drug Administration (FDA) in 2012^4^.

Several studies in the last decade have compared drusen measurements obtained from this software against manual quantification of drusen or similar readouts on different imaging modalities (mainly CFP-based), often showing that measurements across different imaging methods and devices yield different results and are not directly interchangeable^10,11^. One previous work compared drusen volume measurements from two different devices, with similar findings^12^. However, drusen volumes obtained from the FDA-cleared algorithms on Cirrus have not been compared with those from the Spectralis SD (spectral domain)-OCT device (Heidelberg Engineering, Heidelberg, Germany).

Another study found that drusen volume measurements obtained from 145 B- and 15 B-scans in iAMD are similar^13^. Nevertheless, different scan patterns on the same device have not been compared as to their sensitivity and specificity for drusen detection in eAMD and iAMD.

In order to better understand how the use of different devices, softwares and scan patterns might affect drusen volume measurements, we compared all these factors in persons with no AMD, eAMD and iAMD. We included automated drusen volume measures from the FDA-approved software in Cirrus and from two scans (a denser volume scan, 241 B-scans and a less dense volume scan, 25 B-scans) in Spectralis, assessed through a newly developed software, in the MACUSTAR study cohort.

## Methods

We assessed an initial dataset of 258 subjects from the cross-sectional part of MACUSTAR, a multi-center clinical cohort study focused on early stages of AMD^14,15^.

In brief, the major objective of the MACUSTAR consortium is to develop novel clinically validated end-points in the area of functional, structural, and patient-reported outcome measures in patients with iAMD^14,15^. AMD staging (no, early, intermediate and late) for all subjects is reading center–confirmed using multimodal imaging^14,15^. Since drusen assessment was the main focus of this analysis, individuals with late AMD were not included in the study population.

Inclusion criteria, design and goals of MACUSTAR have been previously described^14,15^.

For 10 patients, no imaging data could be retrieved for data analysis due to data management issues. Seven individuals were excluded because of a time gap between Cirrus and Spectralis examinations (> 6 weeks). Two individuals were excluded because of low-quality scans in both eyes (internal Cirrus quality parameter < 6 or internal Spectralis quality parameter < 20 dB). Incomplete date (at least one scan lacking in both eyes) lead to the exclusion of 39 participants. In the analytical population, 20 eyes were excluded due to either a missing scan in one of the three modalities (N = 8), low scan quality (N = 5 Cirrus, N = 3 Spectralis) or artifacts in drusen segmentation (N = 1 for Cirrus, N = 3 for Spectralis), leaving 380 eyes from 200 individuals (no AMD, n = 49 (22.3%), eAMD, n = 25 (13.1%), iAMD, n = 126 (64.6%)) with high-quality, complete data.

This study has been conducted according to the provisions of the Declaration of Helsinki and was approved by local licensing ethic committees of participating countries, including University Hospital Bonn ethics committee (384/17), as listed previously^15^. All participants provided informed consent.

### Imaging

Participants underwent pupil dilation with tropicamide 0.5% and phenylephrine 2.5% after which multimodal imaging according to standard operational procedures was performed^15^. The Spectralis imaging protocol included a 20° × 20° (25 B-scans, approximate distance between scans 240 μm, 4 frames per B-scan) and a 30° × 25° macular volume scan (241 B-scans, approximate distance between scans 30 μm, 9 frames per B-scan)^15^. The SD-OCT Cirrus imaging protocol included a 200 × 200 macular cube (200 B-scans) covering an area of 6 × 6 mm.

### Image grading

Details on the MACUSTAR Image grading have been described previously^15,16^.

In brief, MACUSTAR participants are recruited at 20 clinical sites from seven European countries. Imaging data are graded at the central reading center (GRADE Reading Center, Bonn, Germany) by one junior reader followed by one senior reader grading review according to standardized and predefined grading procedures. For AMD status grading, the dense SD-OCT raster scan was used as the reference imaging modality. The B-scan with the largest possible drusen was preselected and its measurement was used to assess the maximum drusen size, which allowed for classification into small (≤ 63 µm), medium (> 63 µm and ≤ 125 µm) and large (> 125 µm) drusen. RPD were defined as hyperreflective irregularities and elevations above the RPE/BM complex on OCT that had to display corresponding lesions on either infrared imaging or fundus autofluorescence. Prerequisite for grading was a minimum of five individual lesions, each of a diameter of approximately 100 µm.

### Drusen segmentation and quantification

Drusen volume measurements on Cirrus were derived from an established and FDA-approved software, whose measures are repeatable and reproducible^4,5,17,18^.

In brief, in the Cirrus algorithm the observed and expected contours of the RPE layer are obtained by interpolating and fitting the shape of the segmented RPE layer, respectively. The areas located between the interpolated and fitted RPE shapes (which have nonzero area when drusen occurs) are marked as drusen^4,17^.

Drusen quantification on Spectralis is based on OCT layer predictions. BM and RPE layer heights are predicted with a state-of-the-art deep learning model for order-constrained layer regression (predicting layer heights while guaranteeing their correct anatomical order). For the drusen computation, a healthy RPE height is derived from BM and RPE predictions under the assumption that it has a fixed distance to the BM which varies only based on individual physiology and image resolution. The drusen height, required for filtering small false positives, is determined based on connected components in a drusen enface projection. The algorithm on Spectralis was built as an extension of a previously published tool for drusen volume segmentation and is freely available^19–21^. Interestingly, the algorithm on Cirrus and the one on Spectralis adopt a similar method: drusen are computed as the area between the predicted and the computed healthy RPE. In both algorithms, small false positive RPE elevations less than 5 pixels (19.5 μm) high are filtered out^4,20,21^.

To ensure full automation of measurements, neither drusen nor retinal layers segmentation was manually corrected. However, we performed post-hoc quality assurance in both scans and enface projection of drusen segmentation in both devices, ensuring that all scans were fully centered and drusen segmentation maps were plausible. Both algorithms report drusen volume measures both inside a fovea-centered 3- and a 5-mm diameter circle^4^; we only investigated values from the 5-mm circle as they reflect the grid used for Beckmann AMD grading. Pixel-microns conversion was based on the respective formula provided by the Heidelberg Eye Explorer and Cirrus Zeiss software. A previous study showed high comparability between their axial and lateral retinal measurements^22^.

### Statistical analyses

We assessed inter-device and inter-scan differences with intra-class correlation coefficients (ICCs), root mean squared error (RMSE), and mean difference.

Differences between devices were assessed with Wilcoxon paired signed rank test; increases across AMD stages were assessed with the Jonckheere-Terpstra test for ordered variables.

Sensitivity, specificity and area under the curve (AUC) of both algorithms in the population sample were tested with a receiver operating characteristic (ROC) analysis. We tested accuracy of drusen volume measurements in discriminating eyes with any e- and iAMD, as well as subsets with only i- and eAMD, vs controls. We selected drusen volume measurement thresholds maximizing the optimality criterion expressed by the formula below^23^

$$min\left({\left(1-sensitivities\right)}^{2}+{\left(1-specificities\right)}^{2}\right).$$

To assess the relative magnitude of the mean difference in each AMD group, we standardized it by dividing it by the mean average value of the two devices, respectively. We only calculated ICC in the iAMD group due to low variability of drusen volume in no and eAMD, leading to poorly interpretable ICC^24^.

Current algorithms are trained for segmenting the BM-RPE complex; since RPDs are located between the RPE and ellipsoid zone, algorithms may be less consistent in RPD detection and segmentation. For this reason, we stratified ICC by excluding individuals with reticular pseudodrusen (RPD) in iAMD to assess its effect on measures comparability. We reported both consistency and agreement using two-way ICC. In brief, ICC type consistency compares two measures without adding a penalty for a systematic error (x = y + e), contrarily to ICC type agreement(x = y), hence their conjoint assessment is highly informative of measurements’ interchangeability^24^. We visualized differences between measurements from different algorithms and modalities with Bland–Altman plots^25^. Conversion formulae were obtained with Deming regression on the dense Spectralis scan. To assess their accuracy, we randomly split the dataset into approximately 80% of observations for training and 20% for testing and assessed prediction accuracy with mean error and RMSE between converted and observed values. We compared results in the whole iAMD dataset against an optimal iAMD subset based on the Bland–Altman analysis

While continuous drusen volume measures might provide more detailed phenotyping, differences among quantitative measures might be less relevant when considering quantitative cut-offs (e.g., one indicating high risk to progression). Hence, we assessed comparability across modalities in iAMD against a binary cut-off indicating higher progression risk, previously shown at 0.03 mm^3^ in Cirrus^5^. The cut-off was 0.083 mm^3^ in Spectralis and was derived by converting the value of 0.03 mm^3^ with the conversion formula obtained in this paper. All statistical analyses were performed in R (base version 3.4).

## Results

Individuals with AMD were significantly older and had worse VA than individuals without AMD (Table 1). Of the 58 eyes with RPD, 54 (93.1%) had iAMD and 4 (6.9%) had eAMD (Table 1).

**Table 1:** Characteristics of the study population.

	Missing/excluded participants	Analytical population^1^ (N = 200)			Among incl./excl.	Among AMD groups
		No AMD	eAMD	iAMD	P	P
N (%)	48	49 (22.3)	25 (13.1)	126 (64.6)		
Age in years	70.5 (6.9)	68.1 (6.3)	72.0 (6.4)	71.3 (7.5)	0.92	0.021
Sex = m (%)	16 (33.3)	19 (38.8)	6 (24.0)	47 (37.3)	0.403	0.143
BCVA	83.4 (6.13)	86.23 (5.09)	83.20 (5.63)	81.88 (6.75)	< 0.001	< 0.001

*SD* standard deviation, *IQR* interquartile range, *AMD* age-related macular degeneration, *eAMD* early AMD, *iAMD* intermediate AMD, *BCVA* best-corrected visual acuity.

^1^For 180 individuals, both eyes were included; for 20 individuals only one eye was included.

### Sensitivity and specificity

In the dense Spectralis scan, we observed at the selected threshold a specificity of 93.7% and a sensitivity of 91.9% (68.1% for eAMD and 94.7% for iAMD). In the 25 B-scans modality, we observed a higher specificity (97.9%) but a lower sensitivity of 87.0% (36.2% in eAMD and 97.1% in iAMD). When assessing measurements obtained from the algorithm on Cirrus, we observed a specificity of 91.6%. Sensitivity was lower than for both Spectralis scan patterns (14.9% in eAMD, 87.0% in iAMD and 75.1% in the whole sample). ROC curves with AUC of each algorithm for drusen volume assessment and respective thresholds are reported in Supplementary Fig. 1.

### Drusen volume measurements

**Figure 1:**
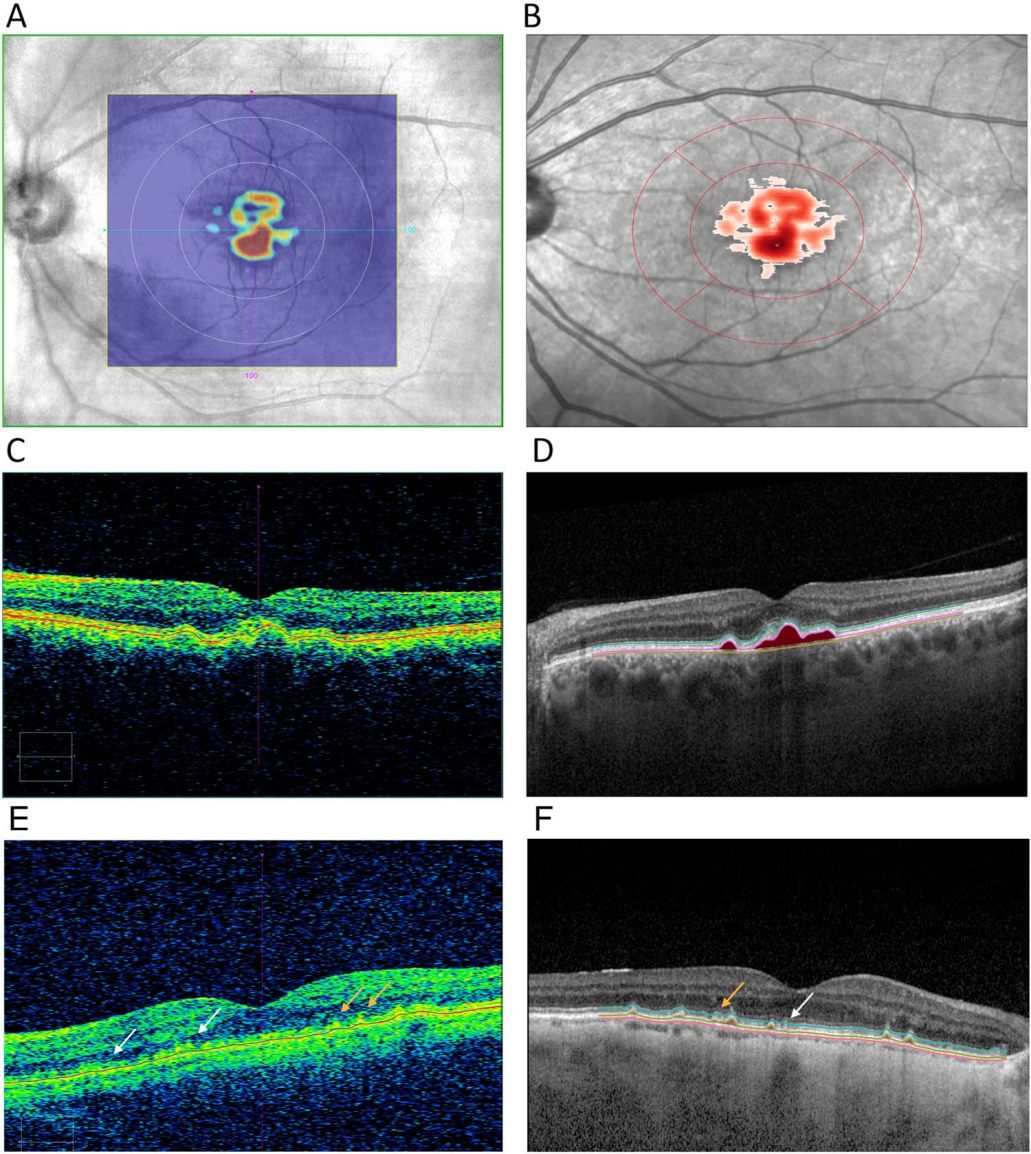
Comparison of Drusen segmentation of the same eye with iAMD, assessed with Cirrus ((**A**) enface projection, (**C**) foveolar B-scan) and Spectralis OCT ((**B**) enface projection, (**D**) foveolar B-scan), showing similar identification. (**E**) (Cirrus) and (**F**) (Spectralis) showing failure to detect (white arrows) or adequately segment (orange arrows) RPE elevations by automated segmentation algorithms in a corresponding macular B-scan.

The denser Spectralis scan yielded on average larger drusen volumes (mean (SD) = 0.082 (0.139)), followed by the less dense Spectralis scan (mean (SD) = 0.0787 (0.135)) and Cirrus (mean (SD) = 0.0398 (0.0857)) (Table 2). We report summary statistics for drusen volume measurements stratified by AMD status in Table 2. All drusen volume measurements showed right-skewed distributions. Differences between drusen volume measurements across all modalities and algorithms were statistically significant (Wilcoxon paired signed rank test p < 0.0001). Drusen volume increased from the no AMD group to early and intermediate AMD in all modalities (Jonckheere-Terpstra test < 0.0001). In all three modalities, mean drusen volume in individuals with RPD (n = 58, of which 54 with iAMD) was slightly lower than in individuals with iAMD without RPD. In Fig. 1 we show a comparison of drusen volume segmentation on the Cirrus (Fig. 1a,c,e) and dense Spectralis scan (Fig. 1B,D) by the respective algorithms.

**Table 2:** Summary statistics for different drusen volume measurements.

	Drusen volume in 5 mm circle (mm^3^)				
	Spectralis, 241 B-scans				
	Mean (SD)	Median (IQR)	10–90%^1^	Range	% = 0^2^
No AMD	0.0002 (0.001)	0 (0)	0–0.00041	0–0.0143	90.5
Early AMD	0.0014 (0.004)	0.0003 (0.008)	0–0.0025	0–0.0188	31.9
iAMD	0.131 (0.158)	0.074 (0.147)	0.035–0.297	0–0.891	0.8
Ret. drusen	0.114 (0.127)	0.068 (0.107)	0.0148–0.255	0–0.672	1.7

	Spectralis, 25 B-scans				
	Mean (SD)	Median (IQR)	10–90%^1^	Range	% = 0^2^
No AMD	0.00002 (0.0001)	0 (0)	0–0	0–0.00125	97.9
Early AMD	0.00102 (0.0027)	0 (0.0007)	0–0.002	0–0.016	63.8
Int. AMD	0.126 (0.154)	0.069 (0.149)	0.002–0.296	0–0.789	2.9
Ret. drusen	0.104 (0.128)	0.056 (0.0972)	0.0119–0.249	0–0.688	1.7

	Cirrus				
	Mean (SD)	Median (IQR)	10–90%^1^	Range	% = 0^2^
No AMD	0.00081 (0.0033)	0 (0)	0–0.0053	0–0.021	91.6
Early AMD	0.00094 (0.0033)	0 (0)	0–0.001	0–0.016	85.1
Int. AMD	0.063 (0.101)	0.022 (0.0702)	0–0.175	0–0.611	13.0
Ret. drusen	0.040 (0.0632)	0.015 (0.0417)	0.0007–0.094	0–0.287	10.3

*SD* standard deviation, *IQR* interquartile range, *AMD* age-related macular degeneration, *eAMD* early AMD, *iAMD* intermediate AMD, *SBP* systolic blood pressure, *BCVA* best-corrected visual acuity.

^1^10–90% indicates 10 and 90% distribution quantiles.

^2^% = 0 indicates percentage of values with undetected drusen (equal to 0).

### Agreement and differences between drusen volume measurements

The mean systematic difference between the dense Spectralis scan and Cirrus was 0.0679 mm^3^ in iAMD and corresponded to 70% of the mean average value (Table 3).

**Table 3:** Measures of agreement, in the whole population and stratified by AMD stage, across different drusen measurements.

Subgroup	Intermediate AMD						
Measure	ICC Cons. [95%CI]		ICC Agreem. [95%CI]		Mean diff. (std.)^3,5^		RMSE^5^
Inter-device comparison^1^	0.807 [0.757–0.847]		0.713 [0.294–0.859]		0.0679 (0.70)		0.107
Inter-scan comparison^2^	0.993 [0.991–0.995]		0.993 [0.991–0.995]		0.0055 (0.04)		0.0188

Subgroup	No AMD		Early AMD		Whole population		
Measure	Mean diff.^5^ (std.) ^3^	RMSE^5^	Mean diff.^5^ (std.)^3^	RMSE^5^	Mean diff.^5^ (std.) ^3^	RMSE ^5^	Agreement4 (kappa)
Inter-device comparison^1^	− 0.0006 (1.22)	0.0037	0.0005 (0.41)	0.0051	0.0424 (0.69)	0.084	89.5 (78.9)
Inter-scan comparison^2^	0.0001 (1.57)	0.0014	0.0004 (0.32)	0.0018	0.0055 (0.04)	0.0149	96.2 (92.4)

*ICC* intra-class correlation coefficient, *Cons.* consistency type, *Agreem.* agreement type, *RMSE* root mean squared error, *Spec.* spectralis, *AMD* age-related macular degeneration, *std.* standardized, *diff.* difference.

^1^Inter-device comparison refers to the 241 B-scans Spectralis modality and Cirrus.

^2^Inter-scan comparison refers to the 241 and 25 B-scans modalities in Spectralis.

^3^The standardized mean value corresponds to mean difference divided the mean average value of the twodevices, respectively.

^4^Assessed against a binary cut-off indicating high progression risk.

^5^Measured in mm^3^.

When comparing drusen volumes between the two algorithms in iAMD, ICC type consistency was higher and more stable than type agreement (ICC [95% CI] 0.713 [0.294–0.859] vs 0.807 [0.757–0.847]), indicating that a systematic error partially accounts for differences in the iAMD group (Table 3). ICC increased when excluding individuals with RPD (n = 184, type consistency ICC [95% CI] 0.831 [0.781–0.871], type agreement ICC [95% CI] 0.752 [0.367–0.879]). When considering only individuals with RPD, the ICC was lower (type consistency ICC [95% CI] 0.645 [0.466–0.774]) and the mean difference (RMSE) were higher than for other subgroups (0.0737 and 0.1116 mm^3^, respectively). The CI of the ICC in individuals with and without RPD did not overlap, indicating a statistically significant difference between the two groups.

Applying the aforementioned high-risk threshold, agreement (kappa) between the dense Spectralis scan and Cirrus was 89.5 (78.9) (Table 3).

Drusen volume measurements of the two Spectralis scans had very high agreement (ICC agreement > 0.99) and the mean difference was 0.0055, corresponding to only 4% of the mean average value.

**Figure 2:**
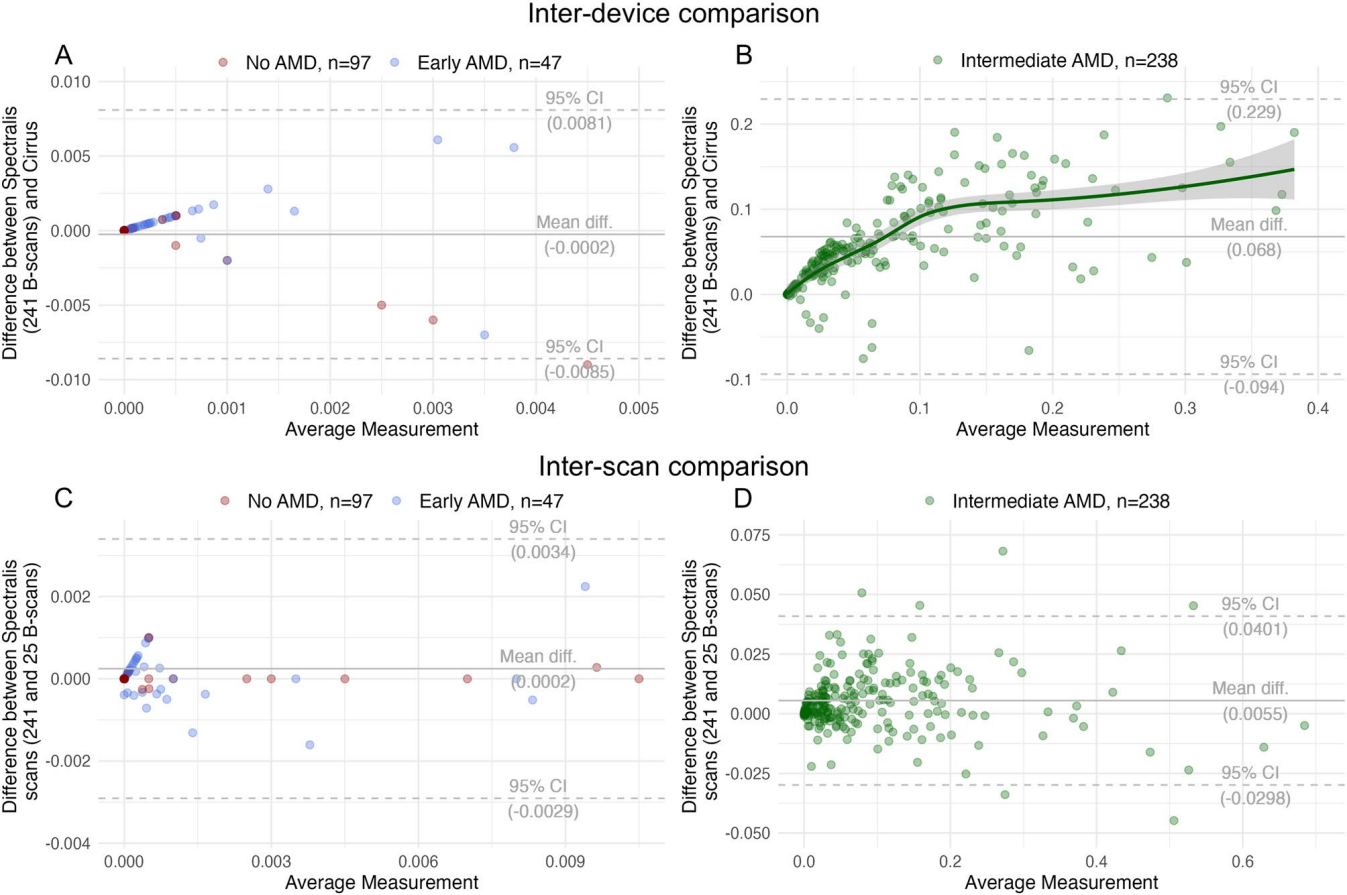
Bland–Altman plot showing differences stratified per AMD stage between the two algorithms (**A**,**B**) and the two scans (**C**,**D**), respectively. A Least Absolute Shrinkage and Selection Operator (LASSO) regression line was added in (**B**) to highlight a linear inter-device trend at lower drusen volume measurements and flattening of the curve with increasingly broader confidence intervals at higher values, indicating lower comparability. *AMD* age-related macular degeneration, *diff.* difference.

In the inter-device comparison of the Bland–Altman plot, we observed, both in e- and iAMD, larger drusen detection on the dense Spectralis scan (in iAMD, n = 223 (93.6%) eyes, the difference between the two modalities was positive) and larger drusen volume measurement on Spectralis at larger average drusen volume measurement on the two devices (Fig. 2a,b). In iAMD, we observed a linear trend between drusen volume measurements in cirrus and the dense Spectralis scan for most data points. At the lower end of drusen volume a small number of participants (n = 13) had larger values on Cirrus and at the upper end, we observed a flattening of the linear trend with an increasingly broader confidence interval, indicating lower comparability. (Fig. 2b). In the inter-scan comparison, we observed smaller drusen measurement on the dense Spectralis scan and a random measurement error between the two modalities (random scatter around the x-axis) (Fig. 2c,d).

### Formulae for algorithms conversion

We identified an optimal dataset with a linear trend between the two measurements consisting of 194 eyes for inter-device drusen volume measurements conversion based on Fig. 2b, i.e. we excluded eyes with measurement obtained from the Cirrus algorithm higher than Spectralis (N = 13) and mean values larger than 0.2 mm^3^, corresponding to a flatter trend with large confidence interval (N = 31). When predicting drusen volume from Cirrus to the dense Spectralis scan in the test dataset, the mean error (RMSE) decreased in the whole iAMD dataset from − 0.0113 (0.0640) mm^3^ to 0.0074 (0.0313) mm^3^ in the optimal dataset. When predicting drusen volume from the dense Spectralis scan to Cirrus, the mean error (RMSE) amounted to 0.0173 (0.0458) mm^3^ in the whole iAMD dataset and − 0.0003 (0.0168) mm^3^ in the optimal dataset.

Data from the two algorithms can be converted in optimal iAMD cases with following formulas: Drusen volume from cirrus algorithm = (drusen volume from Spectralis algorithm) × 0.473 − 0.0091 mm^3^; Drusen volume from Spectralis = (Drusen volume from Cirrus algorithm) × 2.112 + 0.0193 mm^3^.

## Discussion

We present a study systematically comparing drusen volume measurements obtained from two different algorithms on two different SD-OCT devices (Cirrus, Spectralis) and from two modalities at different B-scan density from the same device (Spectralis), as well as evaluating their classification accuracy in no AMD, eAMD and iAMD individuals.

The algorithm using Spectralis images showed a higher sensitivity in both the e- and iAMD groups than the Cirrus algorithm, while specificity was similar. In iAMD, after accounting for a systematic difference, comparability between the two algorithms was good (ICC consistency type > 0.75) and more stable (CI width decreased by 84%). The mean difference in iAMD was 0.0679 mm^3^. The conversion formulae that were provided could be used to collate and compare data from the two algorithms and devices. The formulae were derived in an optimal iAMD dataset; hence they might be less accurate at average drusen volume measurement larger than 0.2 mm^3^ and in case of higher quantification from the Cirrus algorithm.

Comparability between drusen volume measurements from the two devices was lower in individuals with RPD. This might be due to factors both intrinsic to imaging and performance of algorithms for drusen segmentation^26^. In particular, RPD and soft drusen have a different relationship with respect to the RPE, hence current algorithms often fail to accurately segment RPD.

Furthermore, we observed a good agreement between the two devices against a high-risk cut-off in drusen volume in iAMD. This indicates a substantial agreement in detecting individuals at high-risk, which might prove useful in clinical settings to efficiently triage patients^5^.

To the best of our knowledge, a comparison between drusen volume measurements from two different algorithms on Spectralis and Cirrus SD-OCT has not been performed. However, previous studies have observed a systematic difference when investigating other biomarkers (such as retinal thickness or the BM-RPE complex derived with built-in softwares) across the two devices^12,22,27,28^.

Any such differences may be due to differences in image acquisition, resolution and scaling^22,27,28^, device specific softwares (e.g. computational methods, minimal elevation of the RPE necessary to identify drusen) or chosen scan modality (number of A- and B-scans^22^). In our study, the number of significant decimal figures of the two algorithms is different, which might in part account for observed sensitivity differences.

Interestingly, a previous study found comparable retinal thickness measurements from Cirrus and Spectralis utilizing a third-party segmentation algorithm^28^.

Similarly, another study found that differences in drusen volume measurements between two SD-OCT devices decreased when measuring drusen volume with the same third-party software, as compared to measuring drusen volume with in-built algorithms on each device^12^. These findings suggest that differences in software might be more relevant than in hardware; however our study design did not account for dissecting intrinsic image differences against software differences in drusen segmentation.

When assessing drusen segmentation between the dense, 241 B-scans and the less dense, 25-B-scans modalities in Spectralis, we observed almost complete agreement between the two scan patterns in iAMD. Similar findings were observed in a recent study, comparing manually delineated drusen volume with Spectralis in an iAMD cohort between 145 B-scans and 15 B-scans modalities^13^. Our results extend these previous findings, with the observation that a less dense grid might suffice for drusen volume quantification in iAMD but has lower sensitivity in eAMD. This difference is explained by the observation that interpolation of large drusen between the scans might account for a smaller number of B-scans, but medium drusen (between 63 and 125 μm) might occur between B-scans and be more easily missed in less dense scan. In this context, part of the lower sensitivity in Cirrus for eAMD might also be explained by less densely placed B-scans compared to the Spectralis scan (200 vs 241 B-scans, respectively). More studies are needed investigating drusen detection at intermediate B-scan densities, to derive an optimal number of B-scans optimizing examination velocity and detection of smaller biomarkers (such as medium-sized drusen or hyperreflective foci).

Strengths of our study include the well phenotyped sample of participants with no, early and iAMD, the implementation of standardized image acquisition protocols, training of study site personnel, use of a central reading center and implementation of automated image analysis softwares. Limitations include the relatively small sample size, lack of an external validation of our findings and lack of data on repeatability and reproducibility of the Spectralis software while such studies exist for Cirrus^8,17^. However, the high agreement we observed between the two Spectralis scans might be indicative of good reproducibility of its findings.

In conclusion, drusen volume measurements obtained from the two devices and algorithms are not directly interchangeable. In iAMD, accounting for a systematic error largely increased their comparability, possibly allowing for data integration from the two modalities. Presence of RPD further complicated drusen detection and quantification. Comparability between a 25- and 241 B-scans modality was high, but dense scan patterns are required in eAMD. Further research is required to better characterize optimal scan patterns and image analysis softwares for best possible drusen detection and quantification.

## Supplementary Information

Supplementary Figure 1.

## Data Availability

Data are not publicly available. However, the datasets used in the present study can be made available from the MACUSTAR consortium upon reasonable request at dataaccess@macustar.eu.
