# Author Correction: Comparability of automated drusen volume measurements in age-related macular degeneration: a MACUSTAR study report

**DOI:** 10.1038/s41598-023-30360-1

**Published:** 2023-03-07

**Authors:** Davide Garzone, Jan Henrik Terheyden, Olivier Morelle, Maximilian W. M. Wintergerst, Marlene Saßmannshausen, Steffen Schmitz-Valckenberg, Maximilian Pfau, Sarah Thiele, Stephen Poor, Sergio Leal, Frank G. Holz, Robert P. Finger, MACUSTAR Consortium, H. Agostini, L. Altay, R. Atia, F. Bandello, P. G. Basile, C. Behning, M. Belmouhand, M. Berger, A. Binns, C. J. F. Boon, M. Böttger, C. Bouchet, J. E. Brazier, T. Butt, C. Carapezzi, J. Carlton, A. Carneiro, A. Charil, R. Coimbra, M. Cozzi, D. P. Crabb, J. Cunha-Vaz, C. Dahlke, L. de Sisternes, H. Dunbar, E. Fletcher, C. Francisco, M. Gutfleisch, R. Hogg, C. B. Hoyng, A. Kilani, J. Krätzschmar, L. Kühlewein, M. Larsen, Y. T. E. Lechanteur, U. F. O. Luhmann, A. Lüning, I. Marques, C. Martinho, G. Montesano, Z. Mulyukov, M. Paques, B. Parodi, M. Parravano, S. Penas, T. Peters, T. Peto, S. Priglinger, D. Rowen, G. S. Rubin, J. Sahel, C. Sánchez, O. Sander, M. Schmid, H. Schrinner-Fenske, J. Siedlecki, R. Silva, A. Skelly, E. Souied, G. Staurenghi, L. Stöhr, D. J. Taylor, A. Tufail, M. Varano, L. Vieweg, L. Wintergerst, A. Wolf, N. Zakaria

**Affiliations:** 1 Department of Ophthalmology, University Hospital Bonn, Ernst-Abbe Str. 2, 53127 Bonn, Germany; 2 Population Health Sciences, German Center for Neurodegenerative Diseases (DZNE), Bonn, Germany; 3 B-IT and Institute of Informatics, University of Bonn, Bonn, Germany; 4 John A. Moran Eye Center, University of Utah, Salt Lake City, USA; 5 Ophthalmology, Novartis Institutes for Biomedical Research, Cambridge, MA USA; 6 Bayer Pharmaceuticals, Berlin, Germany; 7 Department of Ophthalmology, Universitaetsklinikum Freiburg (UKLFR), University of Freiburg, Freiburg, Germany; 8 Department of Ophthalmology, University Hospital of Cologne, Cologne, Germany; 9 Quinze-Vingts National Ophthalmology Hospital, UPMC-Sorbonne Université, Paris, France; 10 Department of Ophthalmology, University Vita Salute-Scientific Institute of San Raffaele, Milan, Italy; 11 AIBILI Association for Innovation and Biomedical Research on Light and Image (AIBILI), Coimbra, Portugal; 12 Institute for Medical Biometry, Informatics and Epidemiology, University of Bonn, Bonn, Germany; 13 Department of Ophthalmology, Rigshospitalet-Glostrup, Copenhagen University, Glostrup, Denmark; 14 City University London, London, UK; 15 Department of Ophthalmology, Leiden University Medical Center, Leiden, The Netherlands; 16 BAYER AG, Leverkusen, Germany; 17 Novartis Pharma AG, Basel, Switzerland; 18 University of Sheffield, Sheffield, UK; 19 University College London (UCL), London, UK; 20 Fondation Voir et Etendre, Paris, France; 21 Department of Ophthalmology, Porto Medical School, Centro Hospitalar de Sao Joao EPE (Hospital Sao Joao), Porto, Portugal; 22 Department of Ophthalmology Luigi Sacco Hospital, University of Milan, Milan, Italy; 23 Carl Zeiss Meditec, AG, Jena, Germany; 24 Clinical Trial Unit, Department of Ophthalmology, Gloucestershire Hospitals NHS Foundation Trust, Cheltenham, UK; 25 Department of Ophthalmology, St. Franziskus Hospital, Münster, Germany; 26 Ophthalmology and Vision Sciences, The Queen’s University and Royal Group of Hospitals Trust, Belfast, Northern Ireland UK; 27 Stichting Katholieke Universiteit/Radboud University Medical Center (SRUMC), Nijmegen Medical Center, Radbound University, Nijmegen, The Netherlands; 28 Department of Ophthalmology, University of Ulm, Ulm, Germany; 29 STZ Biomed and STZ Eyetrial at the Center of Ophthalmology, University Hospital Tuebingen, Tübingen, Germany; 30 F. Hoffmann-La Roche Ltd, Basel, Switzerland; 31 G. B. Bietti Eye Foundation-IRCCS, Rome, Italy; 32 Ludwig-Maximilians-Universitaet Muenchen (LMU), University Eye Hospital, Munich, Germany; 33 European Clinical Research Infrastructure Network (ECRIN), Paris, France; 34 Centre Hospitalier Intercommunal de Creteil (HIC), Centre Hospitalier Creteil, University Eye Clinic, Paris, France; 35 Moorfields Eye Hospital NHS Foundation Trust (MBRC), London, UK

Correction to: *Scientific Reports* 10.1038/s41598-022-26223-w, published online 19 December 2022

The original version of this Article contained an error in the Methods section, under the subheading ‘Statistical analyses’, where the following sentence has been deleted:

“Furthermore, we calculated the agreement in iAMD based on exceeding a drusen volume cut-off indicating higher AMD progression risk (previously shown at > 0.03 mm^3^ for Cirrus, set on Spectralis by adding the mean difference between the two devices to 0.03).”

The original Article has been corrected.

