# Supplementary material for: Comparability of automated drusen volume measurements in age-related macular degeneration: a MACUSTAR study report
Source: Sci Rep. 2022 Dec 19;12:21911. doi: 10.1038/s41598-022-26223-w (PMC9763254; doi:10.1038/s41598-022-26223-w)
Supplement: Supplementary file 1 — Supplementary Figure 1. [file 41598_2022_26223_MOESM1_ESM.docx]

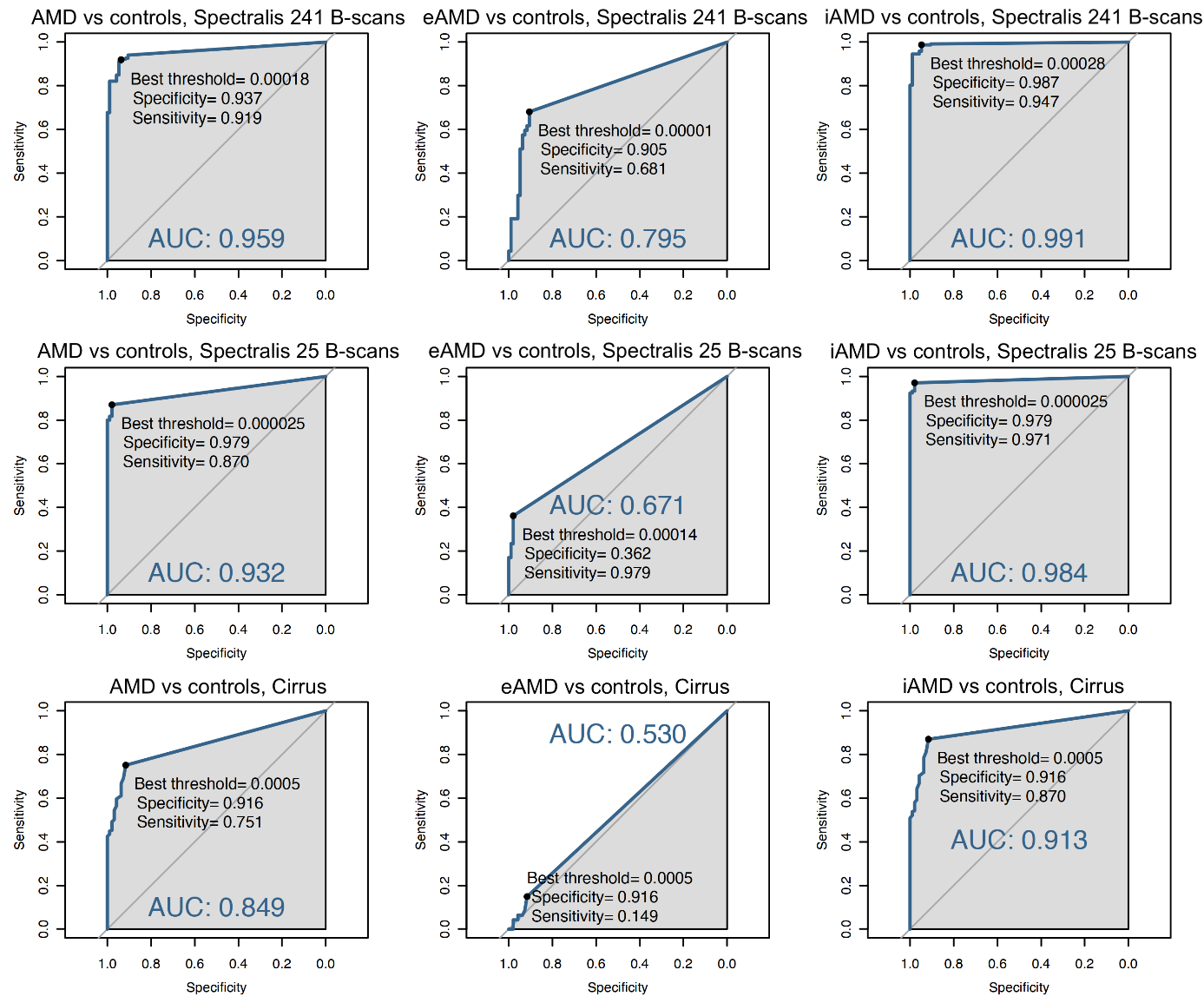


**Supplementary Figure 1,** ROC analysis showing best threshold with respective accuracy and area-under-the-curve of different methods in classifying any, e- and iAMD vs. controls.
